# Geometrical optimisation of core–shell nanowire arrays for enhanced absorption in thin crystalline silicon heterojunction solar cells

**DOI:** 10.3762/bjnano.10.31

**Published:** 2019-01-31

**Authors:** Robin Vismara, Olindo Isabella, Andrea Ingenito, Fai Tong Si, Miro Zeman

**Affiliations:** 1 Photovoltaic Materials and Devices/Else Kooi Lab, Delft University of Technology, Mekelweg 4, 2628CD Delft, The Netherlands; 2 École Polytechnique Fédérale de Lausanne (EPFL), Institute of Microengineering (IMT), Photovoltaics and Thin Film Electronic Laboratory (PV-Lab), Rue de la Maladière 71b, 2002 Neuchâtel, Switzerland

**Keywords:** heterojunction, nanowires, optical modelling, photovoltaics, silicon

## Abstract

**Background:** Elongated nanostructures, such as nanowires, have attracted significant attention for application in silicon-based solar cells. The high aspect ratio and characteristic radial junction configuration can lead to higher device performance, by increasing light absorption and, at the same time, improving the collection efficiency of photo-generated charge carriers. This work investigates the performance of ultra-thin solar cells characterised by nanowire arrays on a crystalline silicon bulk.

**Results:** Proof-of-concept devices on a p-type mono-crystalline silicon wafer were manufactured and compared to flat references, showing improved absorption of light, while the final 11.8% (best-device) efficiency was hindered by sub-optimal passivation of the nanowire array. A modelling analysis of the optical performance of the proposed solar cell architecture was also carried out. Results showed that nanowires act as resonators, amplifying interference resonances and exciting additional wave-guided modes. The optimisation of the array geometrical dimensions highlighted a strong dependence of absorption on the nanowire cross section, a weaker effect of the nanowire height and good resilience for angles of incidence of light up to 60°.

**Conclusion:** The presence of a nanowire array increases the optical performance of ultra-thin crystalline silicon solar cells in a wide range of illumination conditions, by exciting resonances inside the absorber layer. However, passivation of nanowires is critical to further improve the efficiency of such devices.

## Introduction

The implementation of effective and low-cost light trapping schemes is of paramount importance for the development of high-efficiency thin silicon solar cells. The most common approach is the texturing of interfaces, to increase the path length of light inside the absorber. This allows for the use of thinner absorbers, which can decrease manufacturing costs and, in the case of amorphous silicon alloys, reduce the effect of light-induced degradation [1–3]. An alternative approach involves the utilisation of nanostructures that are similar in size to the wavelength of light. This allows for an increase of the electromagnetic (EM) field intensity inside the device, resulting in the enhancement of light absorption [4].

Of particular interest is the employment of elongated nanostructures, such as nanowire arrays. While their nanoscale dimensions can excite various types of resonances of the EM field within the absorber, such as wave-guiding [5–8], cavity modes [5,8–11], Fabry–Perót and whispering gallery modes [12], their characteristic high aspect ratio promotes anti-reflection, allowing for more light to be coupled into the active layer of the solar cell [13–15]. In addition, radial-junction nanowires have the advantage of decoupling absorption and collection, by orthogonalising the path of light with respect to the direction of charge carrier collection [14,16–17]. This aspect allows for the use of lower-quality materials, characterised by short minority carrier diffusion length and/or low absorptivity.

Multiple studies of nanowire solar cells can be found in literature, using different materials: indium phosphide [18–19], gallium arsenide [20–21], zinc oxide [15,22], crystalline silicon [6,8,11–13,16–17,23–34], amorphous silicon alloys [35–37], and recently perovskite [38–41]. In this contribution, the performance of crystalline silicon (c-Si) nanowire arrays is investigated. The study is divided in two parts. First, a proof-of-concept device was realised, consisting of a heterojunction of amorphous silicon on a p-type c-Si nanowire array. The standard manufacturing procedure of c-Si heterojunction solar cells was followed, with the only addition of a cost-effective mask-less reactive ion etching step to create nanowires on the surface of the p-type Si wafer. The resulting 5 × 5 mm^2^ cell exhibits a best-device efficiency of 11.8%, ensuring the feasibility of our proposed device architecture. In the second part, a geometrical study of the nanowire array is carried out, using rigorous optical modelling. An ultra-thin c-Si absorber is employed, to focus the analysis on the effect of nanowires on the propagation of light inside the solar cell. Implied photocurrent densities close to 27 mAcm^−2^ are achieved, for a 2 μm thick c-Si absorber coated with nanowires. The enhanced optical performance, with respect to a flat device, is explained by excitation of resonances both inside the nanowires and in the bulk c-Si absorber. In addition, good angular resilience is displayed, with high implied photocurrent density values (i.e., strong absorption) observed for angles of incidence of light up to 60°, making the proposed solar cell architecture attractive in a wide range of illumination conditions.

## Experimental

### Device manufacturing and characterisation

The nanowire array was manufactured on a p-type mono-crystalline silicon wafer by reactive ion etching (RIE) using a gaseous mixture of SF_6_ and O_2_, followed by standard cleaning, rinsing in de-ionised water and drying of the substrate. In particular, the SF_6_/O_2_ plasma provides a continuous flow of fluorine radicals (F^*^) and oxygen radicals O^*^, which feed two competing chemical reactions: F^*^ and Si react to form SF^4+^ ions, while from the reaction of O^*^ and Si a silicon oxyfluorine (SiO*_x_*F*_y_*) layer is formed. This layer acts as mask against F^*^ etching, but is physically broken by sputtered ions bombarding the surface of the sample. Such effect occurs with higher speed on the horizontal than on the vertical plane, due to the larger angle of incidence of ions hitting the vertical side walls, which leads to a strong anisotropy of the Si etching rate. The process is made mask-less by the precipitation of SiO*_x_*F*_y_* particles, which start the formation of randomly distributed etch pits [42]. These regions become deeper during the process, thanks to the strong anisotropic nature of this RIE etching.

A back-side emitter was formed by phosphorous ion implantation, with energy of 2 × 10^15^ cm^−2^ and dose of 20 keV. Oxidation and annealing were carried out in dry ambient at 850 °C for 90 min, resulting in a sheet resistance *R*_SH_ of 60 Ω/square. Before depositing the coating layers, the silicon wafer with nanowires on top was treated with diluted hydrofluoric acid, to remove the thin native oxide layer from the surface. Plasma-enhanced chemical vapour deposition (PECVD) was used for growing thin-film silicon and silicon alloys layers, to implement surface passivation and front surface field. Intrinsic hydrogenated amorphous silicon (a-Si(i):H), with a thickness equivalent to 30 nm on a flat substrate, was first coated onto the front surface of the wafer on which the nanowires were distributed. Following a hydrogen-plasma treatment, highly transparent boron-doped hydrogenated nanocrystalline silicon oxide (nc-SiO*_x_*(p):H) with 30 nm equivalent thickness was deposited on a-Si:H. For the front electrode, a 100 nm thick transparent tin-doped indium oxide (In_2_O_3_:Sn, ITO) was deposited at low power and low temperature, using radio-frequency (RF) magnetron sputtering. The cell area was defined as 5 mm × 5 mm, using a mask during ITO deposition. The reported equivalent thickness values of thin films on the flat c-Si substrates were characterised via spectroscopic ellipsometry. Finally, using physical vapour deposition, metal electrodes consisting of Ag/Cr/Al were deposited at the front (as patterned grids) and at the rear surfaces of the wafer (full area), with thickness values of 300/30/300 nm and 300/30/2000 nm, respectively.

**Figure 1:**
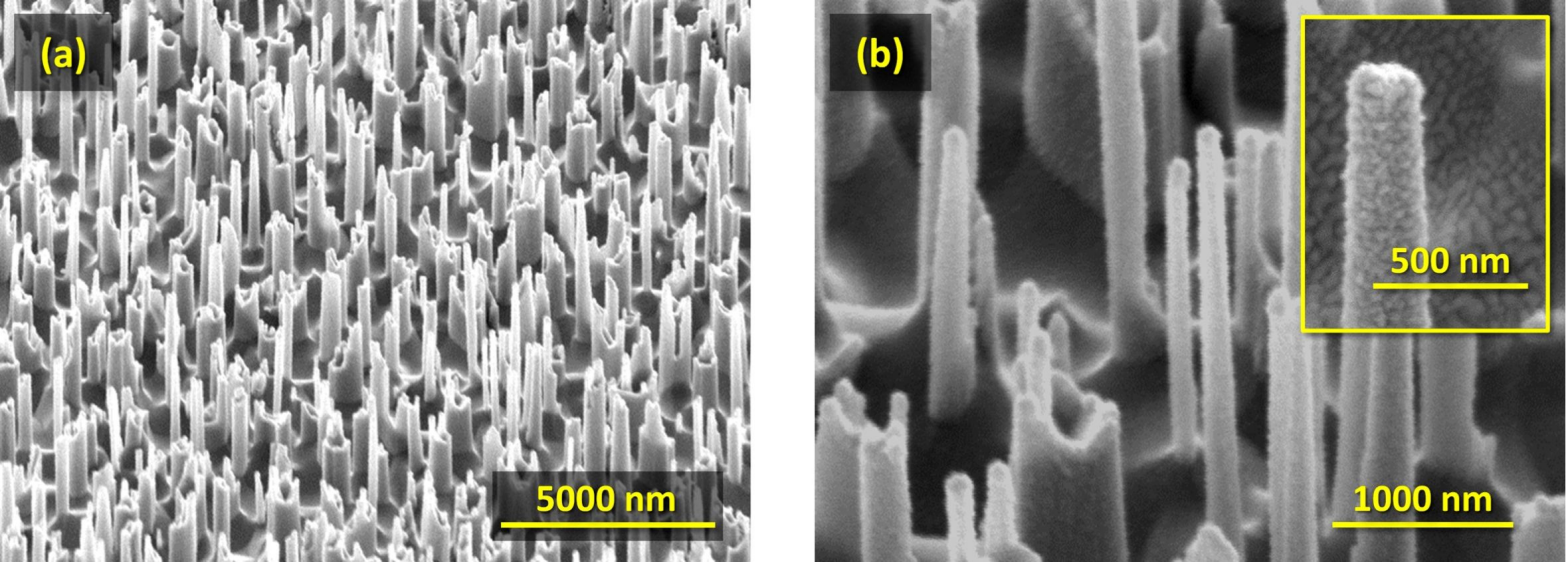
Scanning electron microscopy pictures of (a) bare and (b) coated nanowires on the c-Si substrate. In the inset of (b), the enlargement of a single c-Si nanowire wrapped with supporting layers is depicted, showing excellent coating uniformity.

A Philips XL-50 scanning electron microscope was used for the visual investigation of the nanowires. In Figure 1, pictures of the bare (Figure 1a) and coated (Figure 1b) nanowire arrays are presented. The continuous solar sun simulator Wacom WXS-156S, equipped with a vacuum mask with a 3 mm × 3 mm aperture area, was used to measure the current–voltage characteristics of the fabricated solar cells. The simulator consists of a xenon and a halogen lamp that closely reproduce the spectrum and the intensity of the AM1.5 spectrum [43], which was verified with a c-Si device calibrated at Fraunhofer ISE. For external quantum efficiency (EQE) measurements, the setup used in this work was custom-built. It comprises a Newport illuminator/monochromator, a chopper, a substrate holder (with magnetic pads to hold the probes), and a lock-in amplifier. A calibrated monocrystalline silicon diode with known spectral response was used as reference. The short-current density (*J*_sc_) was determined by a convolution of the measured EQE and the photon flux of the AM1.5 spectrum (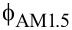). The internal quantum efficiency (IQE) was calculated by dividing the measured EQE by (1 − *R*), where *R* is the reflectance measured by means of a Perkin Elmer LAMBDA 950 UV–vis–NIR spectrophotometer.

### Modelling approach

Simulations of the radial heterojunction c-Si nanowire solar cell were carried out by means of a 3D Maxwell equation solver, based on the finite element method (FEM). The “High Frequency Structure Simulator” (HFSS) was employed [44], which allows for the modelling of thin-film optoelectronic devices with arbitrarily complex geometries [45–52]. To ensure accuracy, accurately measured optical properties (refractive index *n* and extinction coefficient κ) of each material of the structure were used. Simulation results consists of reflection (*R*) and absorption (*A_i_*) in each layer (*i*) of the model, as functions of the wavelength of the incident light. A convolution of the obtained spectral data with the AM1.5 photon flux results in the implied photocurrent density (*J*_ph,_*_i_*) generated (in the active layer) or lost (in supporting layers, or due to reflection):

[1]
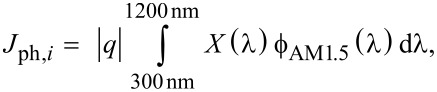


where *q* is the elemental charge, *X* can be either *A_i_* or *R*, and λ is the wavelength of light. Note that only the spectral range between 300 and 1200 nm was considered. In addition, the value of electric and magnetic field inside the structure was exported, to obtain an insight into the propagation of light in the solar cell.

## Results and Discussion

### Device performance

Two series of devices were manufactured: nanowire heterojunction solar cells, with the procedure described in the previous section, and flat references, synthesised through the same process except for the RIE step. The nanowire array has the following (average) dimensions: height 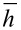 ≈ 2 μm, diameter 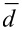 ≈ 200 nm and distance 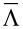 ≈ 800 nm. For each architecture, a total of 48 5 mm × 5 mm solar cells were fabricated, on 4 inch c-Si wafers with an initial thickness of 280 μm.

**Figure 2:**
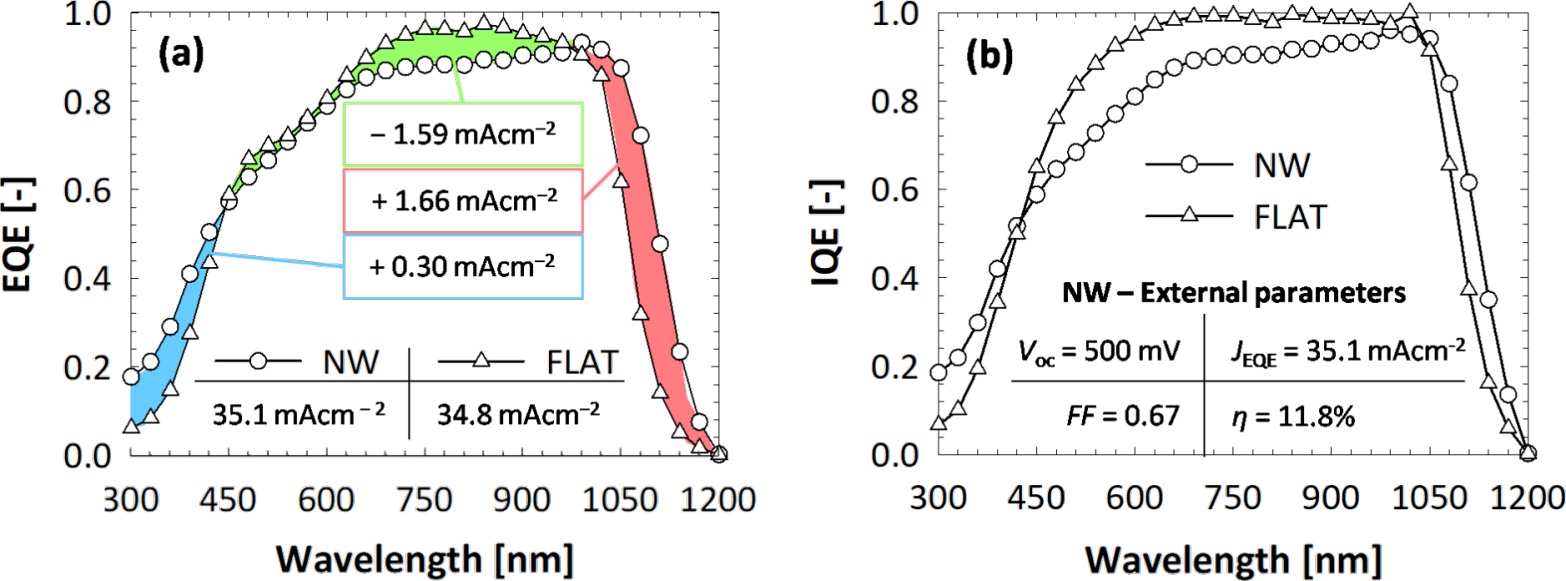
Measured (a) EQE and (b) IQE of the best nanowire heterojunction solar cell (NW) and of the flat heterojunction reference (FLAT). The blue and red areas in (a) indicate the net current gain of the NW structure with respect to the FLAT counterpart at short and long wavelengths, respectively, while the green area indicates the net current loss in the central region of the spectrum of the NW device with respect to FLAT device. The table in (b) reports the external parameters of the best NW device.

In Figure 2a, the (non-biased) EQE of both nanowire and flat devices are depicted. The nanowire solar cell performs better at short and long wavelengths, while its performance suffers in the range between 450 and 950nm. The higher EQE of the nanowire solar cell at short wavelengths (up to λ = 450 nm) can be mainly explained by a better in-coupling of light, promoted by the nanostructure array at the front side. Lower parasitic absorption at the front side can also explain the improvement. This results in a net gain in photocurrent density of 0.30 mAcm^−2^. At longer wavelengths, scattering of photons adds to the anti-reflective effect, resulting in a significant performance increase (+1.66 mAcm^−2^) with respect to the flat device. An additional explanation for the higher performance in these two spectral regions is an increased injection level, due to the same or even higher absorption taking place in less material. The higher carrier concentration results in a performance closer to the radiative limit, which is evidenced by the higher IQE observed at both short and long wavelengths.

On the other hand, the lower EQE in the spectral region of 450–950 nm can be ascribed to a higher charge-carrier recombination (i.e., lower collection efficiency), as highlighted by the IQE curves presented in Figure 2b. While recombination affects the performance across the entire spectrum, at short and long wavelengths this effect is not apparent in Figure 2 since the absorption increase promoted by the nanowires compensates the decreased collection efficiency. Across the 48 individual cells, the low average open-circuit voltage (*V*_oc_ = 495 ± 8 mV) and fill factor (FF = 0.66 ± 0.01) are evidence of high recombination, likely caused by the larger interface area with respect to the flat device. The short-circuit current density (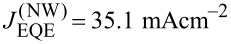), calculated from the EQE measurements, is only slightly higher than the value obtained for the flat reference (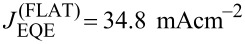), since the absorption gains observed at short and long wavelengths are almost entirely offset by higher charge-carrier recombination. The resulting conversion efficiency is η = (11.5 ± 0.4)%, one of the highest reported values for this type of device [29,32–33].

It can be concluded that the presence of the nanowire array improves the optical performance of the solar cell, namely by promoting very good light in-coupling at the front side and by scattering of photons in the near infrared region of the spectrum, where absorption in c-Si is weak. However, charge-collection efficiency suffers, resulting in low *V*_oc_ and FF and a reduced quantum efficiency, particularly in the visible part of the spectrum. This setback could be avoided by deploying a defect removal etching [53], which would dramatically improve the surface passivation.

### Geometrical study of nanowire arrays

**Figure 3:**
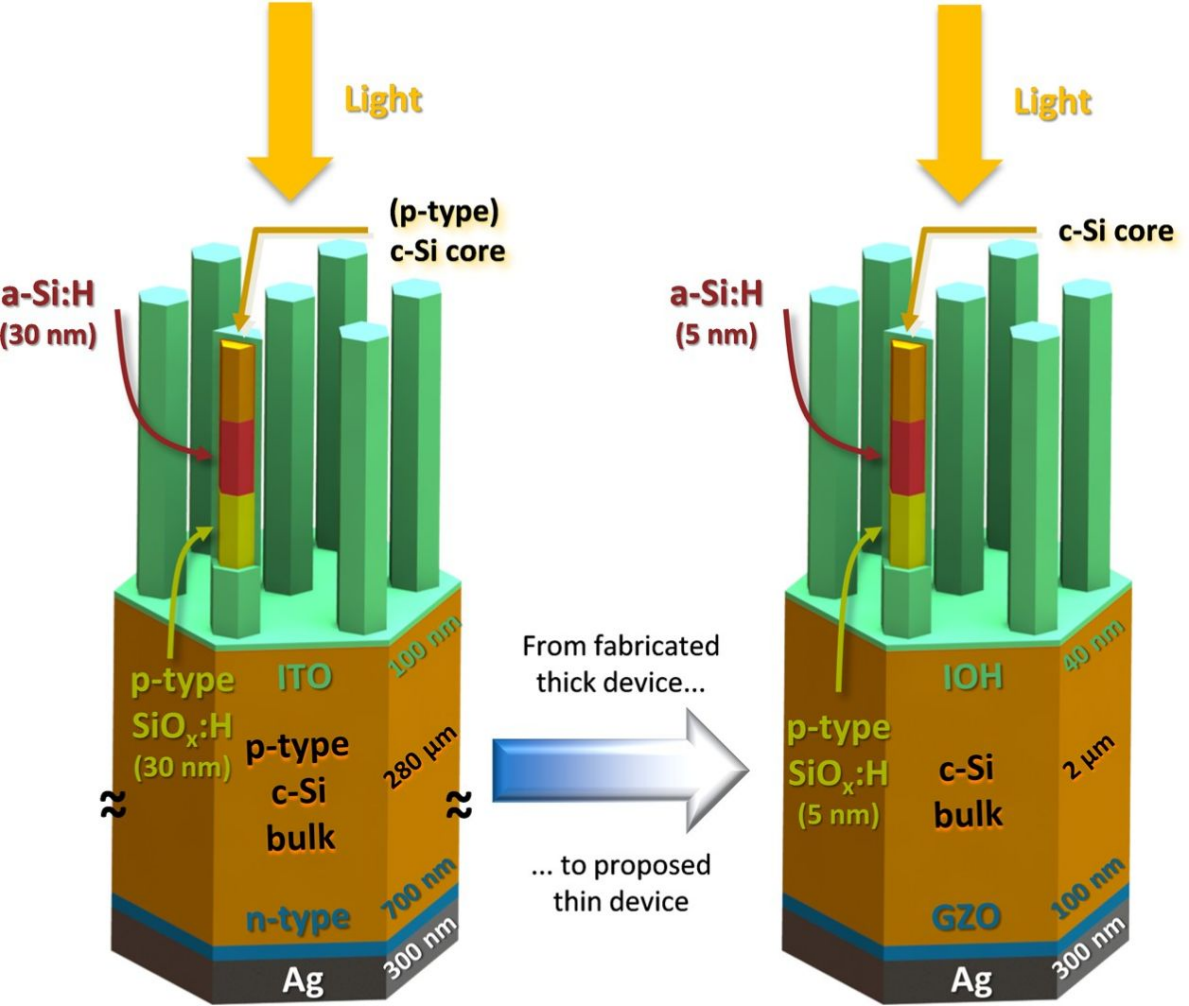
3D rendering of the real device (left) and of the simulation model (right). The differences are: thinner absorber (device: 280 μm, model: 2 μm), thinner and more transparent supporting layers (p-type SiO*_x_*:H + a-Si:H + TCO) at the front (device: 30 nm + 30 nm + 100 nm, model: 5 nm + 5 nm + 40 nm), introduction of a TCO between silicon and metal at the back, in place of the implanted n-type doped silicon layer. The core of one nanowire (c-Si, orange) is presented in both figures, to show the layers that are coating it radially.

To further understand the interaction of light with nanowires, and how the presence of the NW array affects the absorption in the active silicon layer, optical simulations were used. First, a comparison of the absorption is carried out, between a flat reference and a device model endowed with nanowires. The array is assumed periodic (due to modelling constraints) and arranged in a hexagonal lattice. The hexagonal distribution was chosen after a short preliminary study (not reported here for brevity) showed that the hexagonal lattice resulted in slightly higher absorption with respect to square or rectangular ones. This effect was attributed to the larger number of diffraction modes excited by the hexagonal array. Nevertheless, differences between the different arrangements were small, and it is thus assumed that a perfectly random arrangement, such as the one of the manufactured device (Figure 1), would yield similar results. The geometrical properties of the modelled nanowires mirror the dimensions of the manufactured nanostructures: the distance (or period of the array) is Λ = 800 nm, the height is *h* = 2 μm and the cross section is *d* = 200 nm. A depiction of one unit cell of the device model is presented in Figure 3. Appropriately defined periodic boundary conditions ensure the creation of a complete solar cell endowed with an hexagonal nanowire array. The crystalline silicon bulk has a thickness of only 2 μm, to better highlight the effect of the presence of nanowires. At the front side, a stack of a-Si:H (thickness of 5 nm) and p-type nc-SiO*_x_*:H (5 nm) forms the hole-selective contact, followed by In_2_O_3_:H (IOH, 40 nm) in the role of the front transparent conductive oxide (TCO). The three layers uniformly coat both the nanowires and the exposed portion of the bulk. At the back side, the negative contact consists of another TCO, ZnO:Ga (GZO, 100 nm) [47,54], and silver (300 nm). There are a few differences between the manufactured solar cells and the model employed (in addition to the thinner bulk and the periodicity of the nanowire array): (i) To reduce parasitic absorption at the front, the a-Si:H and p-type nc-SiO*_x_*:H layers are significantly thinner, and IOH is preferred to ITO due to its higher transparency and conductivity [55–56]; (ii) at the back, GZO is introduced to improve the reflectivity of the contact. The flat reference employs the same layers (material and thickness) as the nanowire model, the only difference being the absence of the nanostructure array.

**Figure 4:**
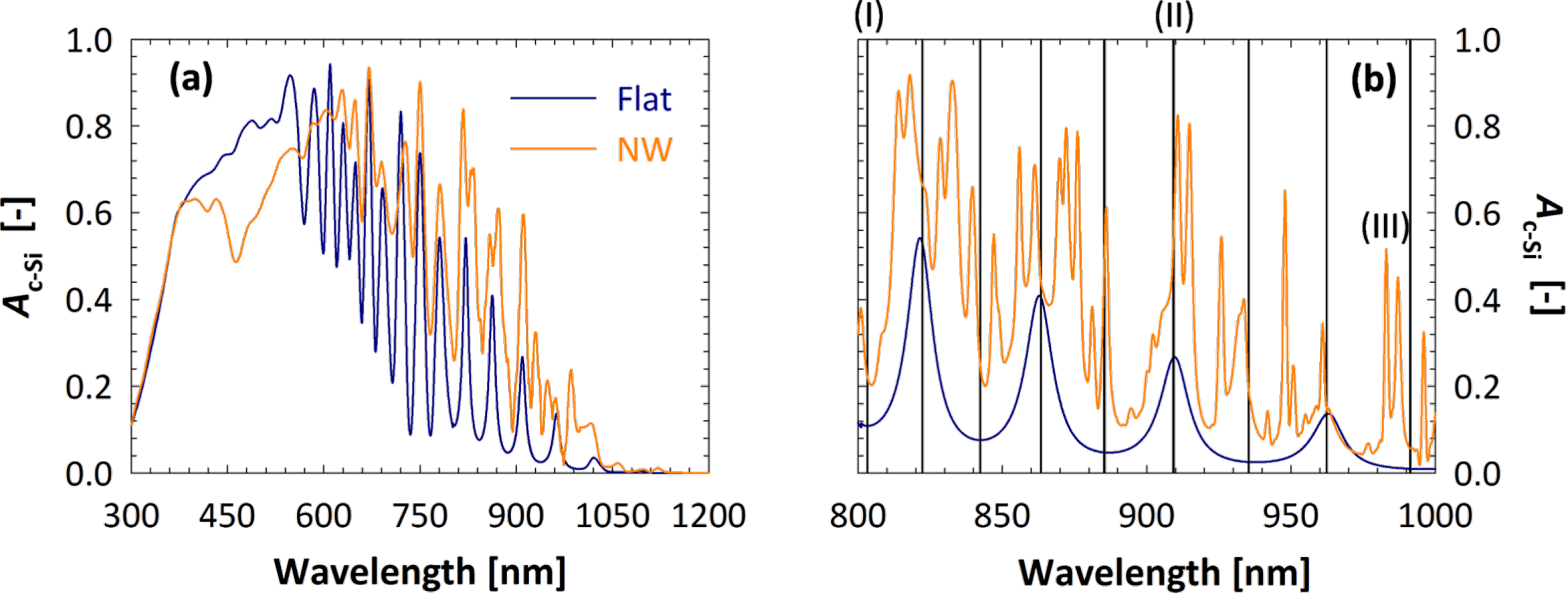
Calculated absorption in c-Si, as function of the wavelength, of the flat reference (FLAT, blue) and nanowire (NW, orange) device models. In (a), the range 300–1200 nm is considered, while (b) focuses on the spectrum between 800 and 1000 nm. Black vertical lines in (b) indicate the position of interference resonances, calculated with Equation 2. The corresponding electric field distributions are presented in Figure 5.

In Figure 4, the calculated absorption in the c-Si layer (*A*_c−Si_) is depicted, for both nanowire device (NW) and flat reference (FLAT). For 400 nm *<* λ *<* 550 nm, the optical performance of the NW model is inferior to the FLAT reference. This result can be explained by the higher absorption in the front layers, particularly a-Si:H, which in the model endowed with nanowires have to cover a larger surface area. In addition, the geometry of the nanowires can result in light being trapped in the front layers and thereby being parasitically absorbed. On the other hand, 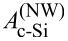 is larger than 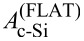 for λ *>* 600 nm. In this region of the spectrum, the absorptivity of supporting layers is weaker, thus the optical performance of the active layer is not strongly affected by their presence. The difference between NW and FLAT architectures is to be ascribed to two factors: (i) The NW solar cell model exhibits lower reflectivity than the FLAT reference, due to the presence of nanowires at the front side; (ii) light propagates differently inside the absorber layer, in particular the absorption spectrum of the NW device displays more (resonance) peaks, as highlighted in Figure 4b for wavelengths between 800 and 1000 nm. In this spectrum range, 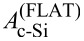 follows the typical profile of a Fabry–Perót interference (F-P), due to the total model thickness being of the same order of magnitude of the wavelength of light. In fact, the position (i.e., the wavelength) of peaks and valleys (black vertical lines in Figure 4b) can be accurately predicted by imposing the condition that the phase difference between primary reflection (air–IOH interface) and secondary reflection (GZO–silver interface) is an integer multiple of π:

[2]
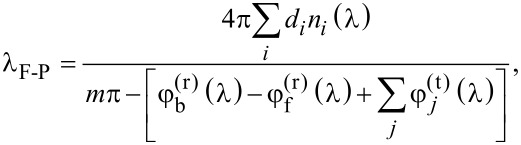


where λ_F−P_ is the wavelength (in vacuo) at which there is constructive or destructive interference between front and back reflected beams. *d_i_* and *n_i_* are the thickness and (wavelength-dependent) refractive index of the *i*-th layer, *m* = 0, 1, 2,…, 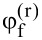 and 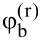 are the (wavelength-dependent) phase shifts taking place when light is reflected at the front and back interfaces, respectively, and 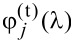 is the (wavelength-dependent) phase shift happening during transmission at the *j*-th interface (between layer *i* and *i* + 1). The absorption profile of the NW model, on the other hand, presents a significantly larger number of peaks. Still the typical shape of F-P interference can be observed, only lifted to higher absorption values due to the diffraction promoted by the presence of nanowires.

**Figure 5:**
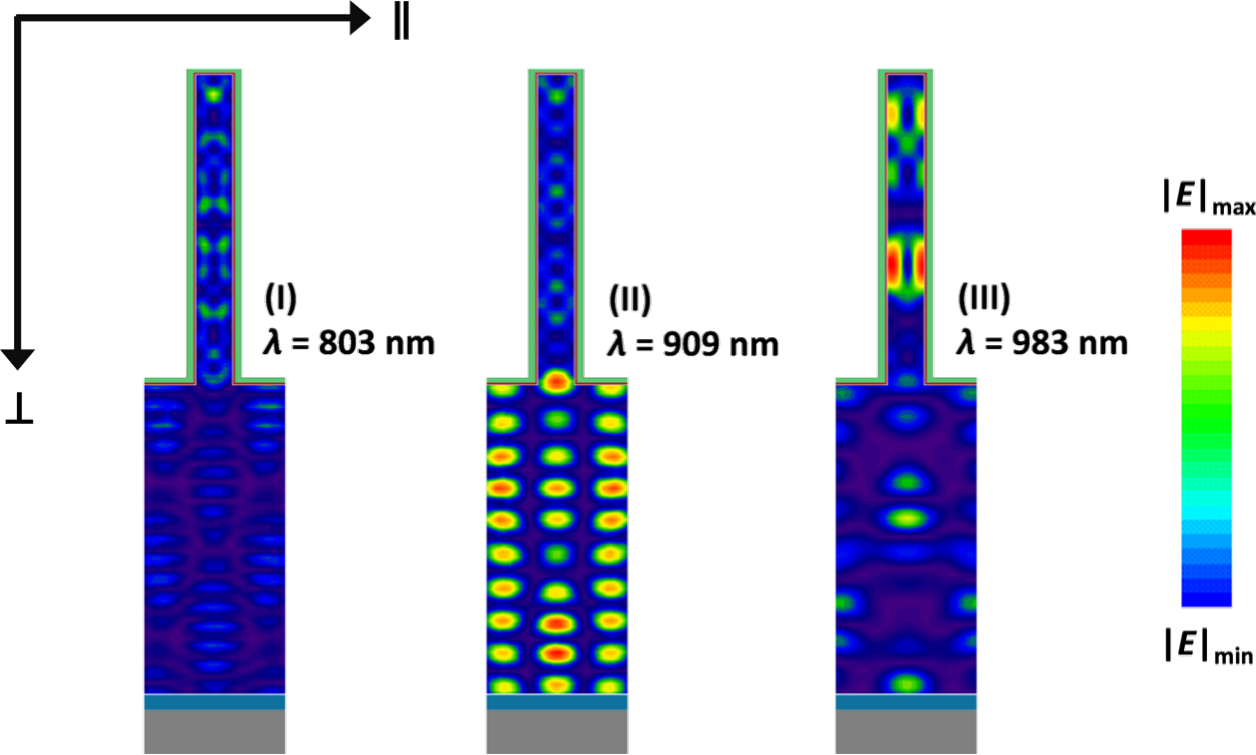
Distribution of the electric field inside the absorber layer of the NW device for three different wavelengths: (I) 803 nm, (II) 909 nm and (III) 983 nm.

The electric field (*E*) distribution inside the device is useful to understand how the propagation of light is affected by the presence of the nanowire array. To this purpose, |*E*| inside the c-Si absorber layer is presented in Figure 5, for three different wavelengths. At λ^(I)^ = 803 nm, Fabry–Perót interference results in a valley in the absorption profile (see (I) in Figure 4). As expected |*E*| is small, with some higher-intensity spots located within the nanowires. This weak guided resonance, combined with the presence of more absorber material, explains that 
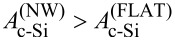
 for λ = 803 nm. On the other hand, at λ^(II)^ = 909 nm several high-intensity regions are observed, particularly in the c-Si bulk. In particular, resonances are excited in both the vertical direction (i.e., the direction of the incident light, 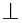), due to F-P interference, and in the horizontal direction (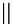), due to interference between diffraction modes inside the silicon layer. The two effects combine to increase the total intensity of the electric field within the absorber layer. This in turn results in a value of absorption, for the NW model, significantly enhanced with respect to the FLAT sample, as shown in (II) in Figure 4. Finally, at λ^(III)^ = 983 nm a peak in 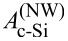 can be seen, while 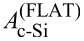 is very low due to being close to a Fabry-Perót minimum. At this wavelength |*E*| is strongly enhanced within the nanowires, which appear to act as cavities for the electromagnetic field. The distribution of |*E*| does not follow the typical F-P interference or diffraction patterns, but can still explain the boost in absorption observed at (III) in Figure 4.

The convolution of *A*_c−Si_ with the photon flux of the solar spectrum (Equation 1) allows for the quantification of the optical performance improvement introduced by the presence of nanowires. The implied photocurrent density generated in the absorber of the NW device (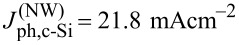) is significantly higher then the value computed for the FLAT reference (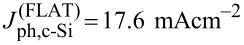), but can be further increased by careful optimisation of the nanowire geometry. To this purpose, the height (*h*) and cross section (*d*) of the nanowires were varied in the ranges of 0–5 μm and 0–700 nm, respectively. The distance between individual nanowires was kept constant at Λ = 800 nm. *h*, *d* and Λ were varied or kept constant within values that are expected to be achievable with the developed RIE process.

**Figure 6:**
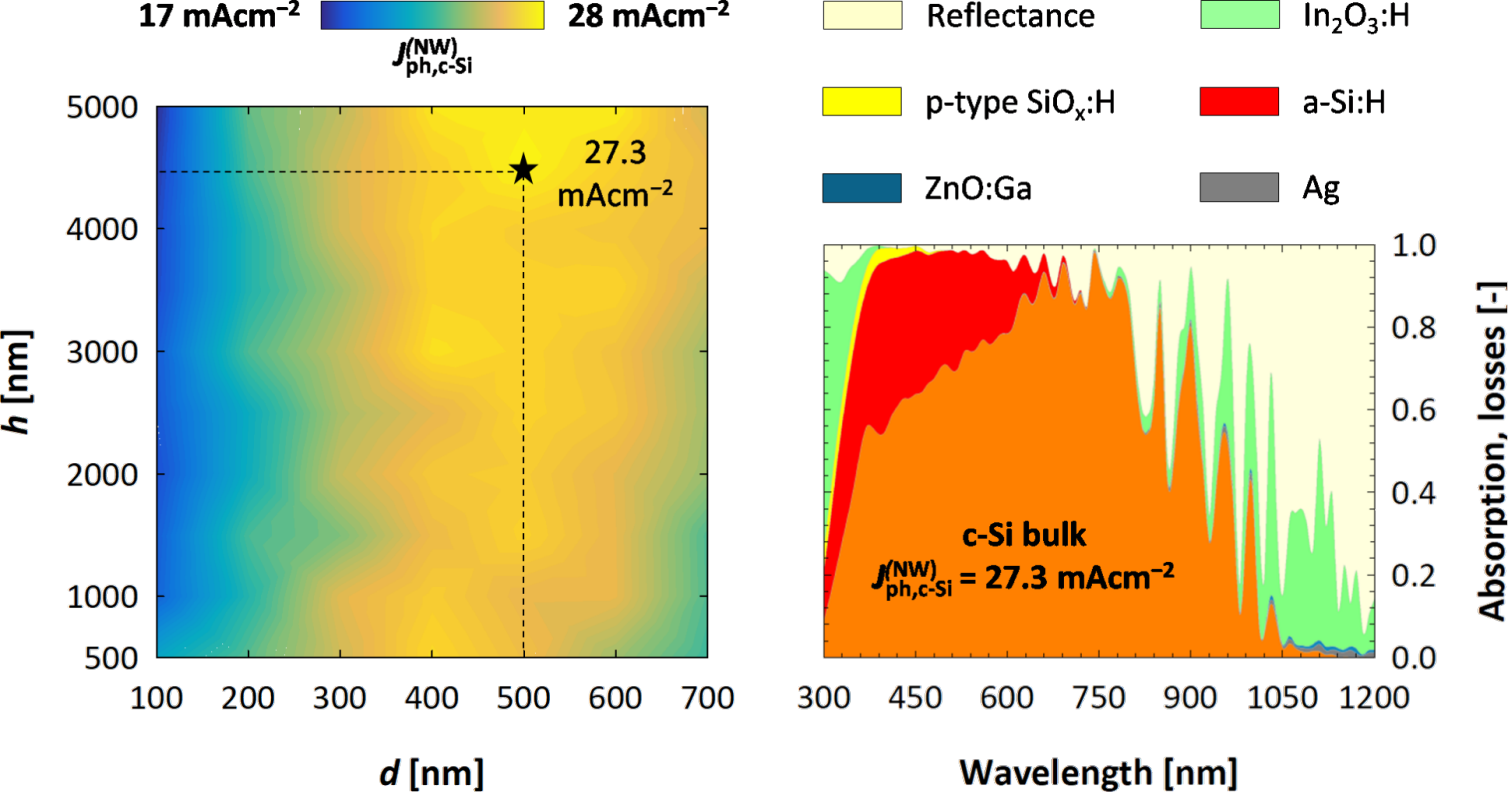
On the left, the implied photocurrent density generated in the c-Si absorber, as a function of cross section (*d*) and height (*h*) of the nanowires. The maximum value (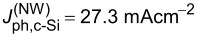) is achieved for *d* = 500 nm and *h* = 4500 nm. On the right, the calculated reflection and absorption in each layer of the model are plotted for the “best” structure.

On the left-hand side of Figure 6, the value of 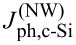 as a function of *d* and *h* is plotted. In Supporting Information File 1, the implied photocurrent density losses, due to reflection and parasitic absorption in supporting layers, are included. It can be observed that an increase in NW height reduces reflectance. This can be expected since (in general) taller nanostructures exhibit better anti-reflection properties. Conversely, losses in the supporting layers increase, since more material needs to cover the taller nanowires. The net result of the two opposite trends is that 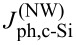 does not exhibit a strong dependence on *h*. In fact, for all values of *d* the difference in 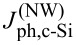 between the best and the worst performing architecture is smaller than 3 mAcm^−2^.

A stronger dependence of performance on the nanowire cross section is observed. On one hand, parasitic absorption losses are (almost) independent on the value of *d*, because the amount of material used in supporting layers does not depend on the NW lateral size. On the other hand, reflectance losses are significant for narrow nanowires (*d <* 200 nm), decreasing sharply until reaching a minimum between 400 and 500 nm. For larger values of the cross section (*d >* 500 nm), reflection losses become larger again. This behaviour can be explained as follows: When *d* is too small the space between individual wires is wide, reducing the amount of light that hits the NWs and can be absorbed. By increasing the cross section, a larger portion of the incident radiation will hit the nanostructures and thereby be absorbed. If *d* becomes too large, however, more and more light is reflected by the top surface of the nanowire, thus increasing total reflection. Anttu et al. suggest another possible explanation for the optimal cross section value [19]. In their work on III–V semiconductors nanowire arrays, they observed the presence of optimum, bandgap-dependent nanowire diameter values. They associate the calculated optima with specific, diameter-tunable nanophotonic resonances, implying that for a specific semiconductor material an optimal value of the diameter can be found that maximises absorption owing to the excitation of resonant modes at specific wavelengths.

The final result is that the 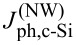 achieves its maximum when reflection is at a minimum (i.e., for *d* = 400–500 nm). The highest performance is achieved for a solar cell model with *d* = 500 nm and *h* = 4500 nm, reaching an implied photocurrent density value of 27.3 mAcm^−2^. Further analysis of the optical losses of the “best” structure (Figure 6, right) reveal that a significant amount of light is parasitically absorbed in the intrinsic a-Si:H layer. On the other hand, it is well known that a-Si:H layers in heterojunction devices do contribute to the charge generation, thus adding to the short-circuit current density [57]. This effect can be noted in Figure 2a, where the EQE is higher than the absorption depicted in Figure 6, and could be quantified with a rigorous electrical simulation, which is beyond the scope of this work. Nevertheless, the choice of a more transparent passivating layer could result in significant increase of absorption, particularly at short wavelengths (λ *<* 600 nm), and in an increase of 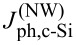 up to 4 mAcm^−2^. It must be noted that the best implied photocurrent density value achieved (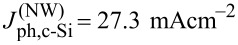) is significantly smaller than what was measured for the manufactured NW device (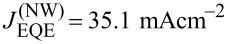). This can only be ascribed to the significant difference in thickness, which in the case of the modelled structures is more than 100 times smaller (2 μm) than that of the nanowire solar cell (280 μm).

**Figure 7:**
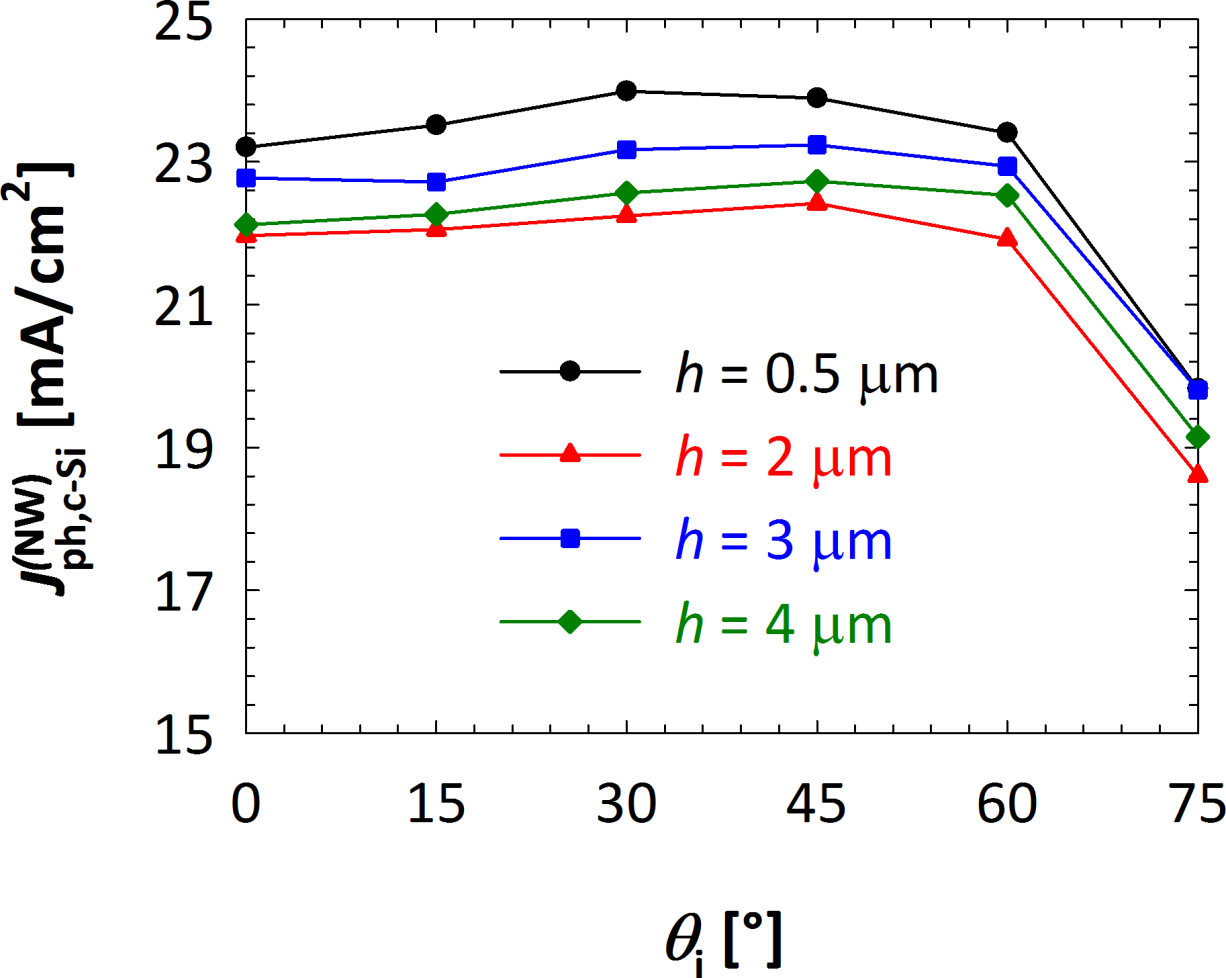
Calculated implied photocurrent density inside the c-Si layer as a function of the angle of incidence of light, for different values of the nanowire height and fixed period (Λ = 800 nm) and cross section (*d* = 200 nm). For clarity of the picture, only selected results are included (*h* = 0.5, 2, 3 and 4 μm).

Finally, the effect of the angle of incidence of the light (θ_i_) was studied. For different heights and constant values of the period (Λ = 800 nm) and cross section (*d* = 200 nm), θ_i_ was varied between 0° and 75°. Results (expressed in terms of 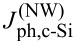) are presented in Figure 7. The optical performance remains fairly constant over a wide range of the angle of incidence. Only for very large angles (θ_i_ *>* 60°) a decrease in *J*_ph,c−Si_ is observed. Device models with different nanowire heights all follow this trend, showing that nanowire solar cells can efficiently absorb light over a wide range of illumination conditions, independent on the size of the NWs. In addition, the performance for different values of *h* is similar within the entire range of angles of incidence (0° *<* θ_i_ *<* 60°). These results are consistent with the findings of the height sweep in the case of perpendicular incidence (Figure 6a) for which it was shown that *h* has little to no effect of the calculated implied photocurrent density of the absorber.

## Conclusion

Nanowires have the potential for improving the optical performance of ultra-thin (ca. 2 μm) c-Si solar cells. The fabricated heterojunction c-Si NW-based solar cell displayed enhanced absorption of light. However, the electrical performance suffered, limiting the final conversion efficiency to (11.5 ± 0.4)%. The optical simulation of NW-based solar cells demonstrated that NWs amplify Fabry–Perót resonances and, at the same time, excite wave-guided modes inside the thin absorber layers. A study of the effect of the NW geometrical parameters on light absorption was carried out. For a given periodicity (Λ = 800 nm) of the NW array and thickness of supporting layers, the optimal NW dimensions were determined resulting in 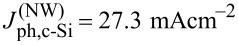. It should be noted that an optimisation of the array periodicity could further improve the optical performance, particularly by choosing a value of Λ closer to the band-gap wavelength of c-Si (λ_BG_ = 1107 nm) [52,58–59]. However, the manufacturing of such device would require abandoning the proposed mask-less approach in favour of a (potentially) more expensive lithography process and was thus not investigated in this work. Finally, it was observed that NW-based solar cells maintain high performance over a wide range of angles of the incidence light, up to 60°.

## Supporting Information

Supporting information includes: (i) measured reflection of the FLAT and NW devices, to complement the data presented in Figure 2; (ii) the implied photocurrent density losses, due to reflection and parasitic absorption, as function of height and cross section of the nanowires. The two pictures are complementary to the data presented in Figure 6 of this manuscript.

File 1 Additional experimental data.
