# Supplementary material for: Geometrical optimisation of core–shell nanowire arrays for enhanced absorption in thin crystalline silicon heterojunction solar cells
Source: Beilstein J Nanotechnol. 2019 Jan 31;10:322–31. doi: 10.3762/bjnano.10.31 (PMC6369979; doi:10.3762/bjnano.10.31)
Supplement: File 1 — Additional experimental data. [file Beilstein_J_Nanotechnol-10-322-s001.pdf]

BEILSTEIN JOURNAL OF NANOTECHNOLOGY

Supporting Information

for

Geometrical optimisation of core–shell nanowire arrays for enhanced absorption in thin crystalline silicon heterojunction solar cells

Robin Vismara, Olindo Isabella, Andrea Ingenito, Fai Tong Si and Miro Zeman

Beilstein J. Nanotechnol. **2019,** *10,* 322–331. doi:10.3762/bjnano.10.31

Additional experimental data

License and Terms: This is a supporting information file under the terms of the Creative Commons Attribution License (http://creativecommons.org/licenses/by/4.0). Please note that the reuse, redistribution and reproduction in particular requires that the authors and source are credited.
The license is subject to the *Beilstein Journal of Nanotechnology* terms and conditions: (https://www.beilstein-journals.org/bjnano)

Reflectance of manufactured devices

In Figure S1, the measured reflection spectra of the FLAT and NW devices are presented. The reflectance of the NW cell is significantly lower than for the FLAT reference, particularly at short and long wavelengths.

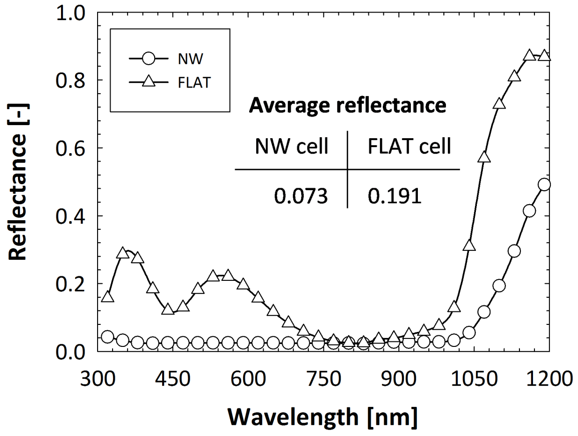

Figure S1: Measured reflectance spectra of the NW and FLAT solar cells.

Calculated losses as function of nanowire height and cross-section

In Figure S2, the losses in implied photocurrent density due to reflection and parasitic absorption are presented.

In the case of reflection, better performance (i.e. smaller losses) is displayed by taller nanowires (larger h) and for cross-section (Λ) values between 300 nm and 500 nm. The global maximum ($-9.4\,\mathrm{mA/cm^2}$) is achieved at $(h,\Lambda) = (5000\,\mathrm{nm}, 400\,\mathrm{nm})$.

Looking at parasitic absorption losses, they show a strong dependence on nanowire height (reduced losses for smaller values of h) and a weaker dependence on the cross-section. The global maximum ($-4.3\,\mathrm{mA/cm^2}$) is observed at $(h,\Lambda) = (500\,\mathrm{nm}, 100\,\mathrm{nm})$.

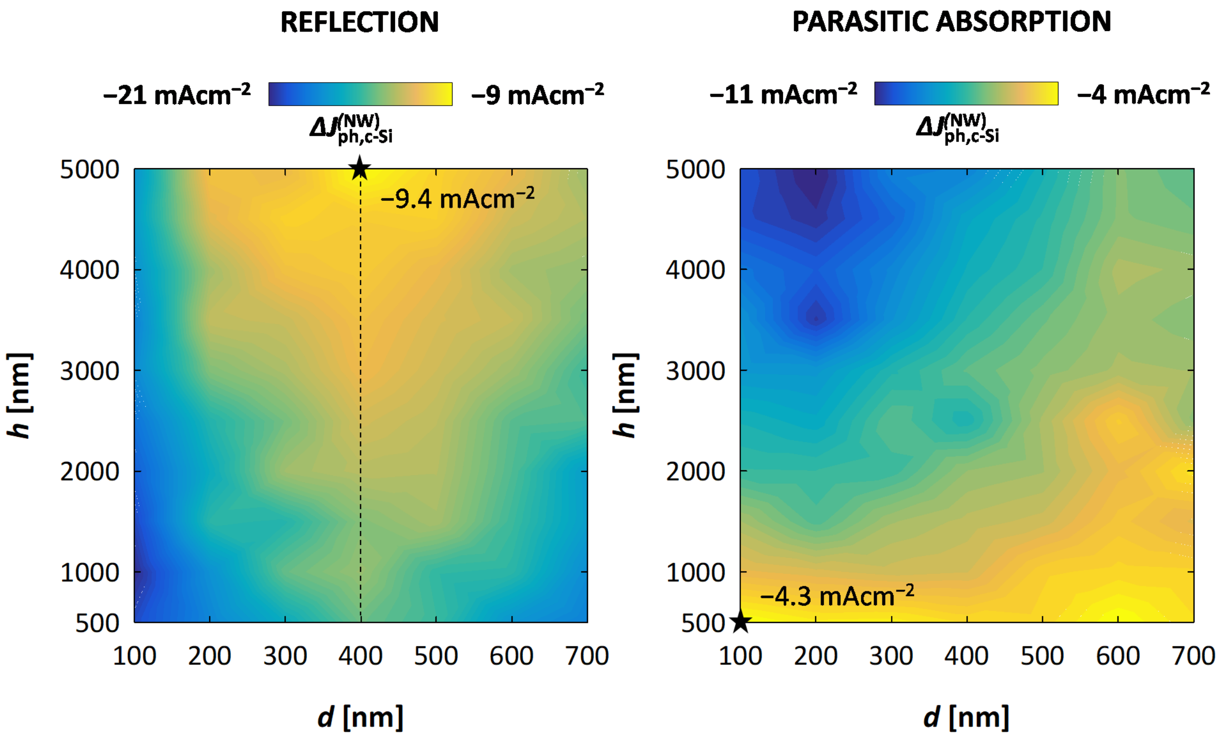

Figure S2: Implied photocurrent density losses due to reflection (left) and parasitic absorption (right).
